# An Assessment of the Psychometric Properties of the GHQ-12 in an English Population of Autistic Adults Without Learning Difficulties

**DOI:** 10.1007/s10803-020-04604-2

**Published:** 2020-07-11

**Authors:** Emese Mayhew, Lucy Stuttard, Bryony Beresford

**Affiliations:** Social Policy Research Unit, Alcuin Block B, University of York, Heslington, York, YO10 5DD UK

**Keywords:** General health questionnaire, Psychometric properties, Autistic adults, Confirmatory factor analysis, Rasch analysis

## Abstract

Valid and reliable tools to measure mental health are a key requirement to developing a robust evidence base on mental health difficulties and autism. There are several reasons why mental health measures developed for the neurotypical population may not be valid and reliable when used with autistic adults. Using data collected from a national evaluation of community-based, specialist autism provision in England, this study assessed the psychometric properties of the General Health Questionnaire (GHQ-12) in a population of autistic adults without learning difficulties. We examined the measure’s acceptability, reliability and internal construct validity. The GHQ-12 was found to have good psychometric properties in this population. This provides first evidence that this measure can be used with autistic adults without LD.

## Introduction

There is strong and growing evidence that autistic adults without learning disabilities (LD) are more likely to be diagnosed with chronic mental health problems, particularly anxiety and depression, than the general population (Croen et al. 2015; Buck et al. 2014; Hofvander et al. 2009; Joshi et al. 2013; Lugnegard et al. 2011).

In order to understand and identify the best ways to respond to mental health difficulties experienced by autistic adults, clinicians and researchers need reliable and valid measures of mental health. For example, they are necessary for establishing the epidemiology of mental health difficulties and evaluating interventions. However, the mental health measures being used for such work were developed for use with neurotypical populations [for whom their psychometric properties (e.g. reliability, validity) are well-established]. It is only recently that questions have begun to be asked about whether these measures of mental health are equally valid and reliable when used in studies of autistic adults. This reflects wider concerns about outcome measurement within the research and practice communities (Ayres et al. 2018).

This paper reports an evaluation of the psychometric properties of a particular measure of mental health, the General Health Questionnaire (GHQ), and specifically the twelve item GHQ-12. The GHQ-12 is perhaps one of the most long-standing and frequently used measure of mental health across the world, including studies of autistic adults (Moss et al. 2015; Picardi et al. 2015). To date, however, its psychometric properties when administered to autistic adults have not been assessed. As with any measure, this is concerning because there are a number of reasons why, potentially, the GHQ-12 may perform differently with this population.

Current discourse considers autism as part human neurodiversity, characterised by a blend of cognitive strengths and weaknesses across the domains of (social) communication, sensory processing, motor skills, reflexive thinking and self-regulation (Robertson 2010). It is not known, however, whether neuropsychological differences may affect the way people respond to the GHQ-12. In addition, recent research has found that difficulties with social interactions often experienced by autistic people might be partly caused by a higher prevalence of alexithymia in the autistic population (Hill et al. 2004). Alexithymia refers to a personality trait where a person finds it difficult to recognise, express and recall his or her own emotions and moods resulting in a reduced emotional understanding of self and others (Poquérusse et al. 2018). Again, there are potential implications when considering self-reported measures of mental health. Therefore, the complex and diverse neurological profile of this population might lead to different interpretations of the GHQ-12, especially items which require the respondents to assess their emotional state. As a result, there might be systematic differences in the psychometric properties of the GHQ-12 in a population with autism compared to the general population.

The aim of this study was to utilise a dataset collected during the course of a national study evaluating models of delivering community-based autism-specialist services to autistic adults without learning disabilities in England (Beresford et al. in press) to examine the psychometric properties of the GHQ-12. Specifically, we assessed its acceptability, factor structure, internal consistency and reliability, and responsiveness to change.

### About the GHQ-12

The General Health Questionnaire (GHQ) was designed as a self-administered screening test for detecting, and measuring, minor psychiatric disorders or psychological distress (Goldberg 1978; Goldberg and Williams 1988). The 12-item version (GHQ-12) is widely used in clinical practice and research (Goldberg and Williams 1988; Richardson et al. 2007; Henkel et al. 2003; Jones et al. 2006). It has been found to have high internal consistency and good retest reliability (Werneke et al. 2000). Its validity has been proved by its linear associations with independent clinical assessments and it has been shown to have good discriminatory power (Goldberg et al. 1997).

The GHQ-12 consists of six positively phrased (PP) and six negatively phrased questions (NP) (Appendix 1). A four point Likert-type response format is used to answer the 12 items. There are three different methods of scoring: Standard (binary scored as follows: 0011), Likert (0123) and Chronic (binary scored as follows: PP: 0011; NP: 0111). It is generally used as a unidimensional measure producing a summated score used as an indicator of severity of psychological distress that can be tracked over time. The score can, however, also be converted into a binary indicator of presence/absence of mental illness by using a cut-off point; for example, the population mean (Shelton and Herrick 2009; Goldberg 1978). Some research indicates that the scoring method has a substantial impact on model estimation (Campbell and Knowles 2007; Crockett et al. 2009; Hankins 2007).

Some studies have presented evidence of the scale comprising two or three factors or domains (Martin and Newell 2005; Shevlin and Adamson 2005; Graetz 1991; Smith et al. 2010), identified as representing psychological constructs such as ‘social dysfunction’, ‘anxiety/depression’ and ‘loss of confidence’ (Graetz 1991; Campbell and Knowles 2007; Picardi et al. 2001). However, others, notably Hankins (Hankins 2008a, b), suggest that the factorial structure reported by some researchers is caused by the scale’s use of positively and negatively worded questions (the so-called ‘method effect’), with negatively phrased questions causing response bias. To test this Hankins performed Confirmatory Factor Analysis (CFA) entering the GHQ-12 as a unidimensional latent construct with correlating error terms on the negatively worded questions; from hereon referred to as the Response Bias Model. He found that the fit of this model outperformed previously established three-factorial (Gao et al. 2004; Graetz 1991; French and Tait 2004) and unidimensional (uncorrelated error term) models. Romppel et al. (2013) validated Hankins’ Response Bias Model on a representative sample of the German general population. Overall, once the underlying causes of the apparent anomalies are understood, it is widely accepted that the GHQ-12 can be treated as a unidimensional measure of psychological distress.

## Methods

### Study Population

Our study population consisted of autistic adults without learning disabilities recruited to a national project evaluating adult, community-based, multi-disciplinary autism-specialist services in England that provide autism diagnostic assessments and coordinate or deliver autism-specialist care and support to those diagnosed by the service and to autistic adults previously diagnosed in childhood or earlier in their adult lives (Beresford et al. in press). Such provision was recommended by national clinical guidance in response to the gap in care and support for autistic adults not eligible for support from statutory health and social care learning disability services (NICE 2012). These are community services (based in community mental health trusts), rather than tertiary, inpatient or residential mental health provision. The overall aim of the project was to identify the features, or characteristics, of service organisation, structure, delivery or practice which impact on service user outcomes. The overarching project objective was to enable evidence-informed decision-making about the commissioning and development of services for autistic adults without LD. Nine services were involved in the study, the majority (6/9) accepted referrals of those already diagnosed with autism as well as those not yet diagnosed with autism.

This paper reports a secondary analysis of the baseline data in order to assess the psychometric properties of the GHQ when used with autistic adults. We also report on autistic adults’ views about the measure and its responsiveness to change. All individuals attending their first diagnostic or needs assessment appointment at entry into a service were approached regarding participation in the study. In three services this included individuals referred to the service for a diagnostic assessment but, due to where they lived, were not eligible for post-diagnosis support. Baseline measures (T0) were completed at the time of recruitment and followed up at 6, 12, 18 and 24 months. Measures completed at each time point included: GHQ-12, a global measure of quality of life [WHO-QoL BREF (WHO 1998)], and health-related quality of life [EQ-5D-5L (EuroQol Group 1990)]. Individuals chose to complete baseline measures in clinic following their first assessment appointment, or at home. Paper and electronic versions were available and assistance with completion by a local member of the research team was available.

Recruitment took place between February 2016 and December 2017. Services varied in the period of time recruitment was open; it ranged from 12 to 18 months. The recruitment rate was 57.2% (422/741). Over a quarter (*n* = 114) recruited became ineligible for the study after the diagnostic assessment did not diagnose autism. Those remaining in the study (*n* = 308), comprised 220 individuals not previously diagnosed with autism, and 88 already diagnosed with autism.

The demographic and mental health characteristics of our population is described in Table 1. Further detail on the study sample is presented elsewhere (Beresford et al. in press).

**Table 1:** Sample characteristics, percentages and (numbers)

Variable	Statistic	Total (*n* = 308)	Male (*n* = 184)	Female (*n* = 114)
Age	Mean Mode (sd dev)	30.56 26.0 12.18	31.66 27.5 12.59	29.1 25.0 11.52
Age groups	≤ 21 22–33 34–43 44–53 ≥ 54	33.4% (103) 34.4% (106) 11.7% (36) 14.3% (44) 5.5% (17)	33.2% (61) 31.0% (57) 10.9% (20) 19.6% (36) 5.4% (10)	34.5% (39) 39.8% (45) 13.3% (15) 6.2% (7) 6.2% (7)
Education	No school leaving qualificatons School leaving qualifications (e.g. GCSE/O-level) Further education Higher education qualification (e.g. university degree)	10.1% (31) 25.8% (79) 36.3% (111) 27.8% (85)	12.5% (23) 26.6% (49) 35.3% (65) 25.5% (47)	7.1% (8) 24.8% (28) 37.2% (42) 31.0% (35)
GHQ-12 score	Likert mean (sd dev)	18.21 (7.36)	17.86 (7.45)	18.82 (7.29)
	Standard mean (sd dev)	5.37 (3.84)	5.58 (3.75)	5.22 (3.49)
	Chronic mean (sd dev)	7.77 (2.77)	7.74 (2.83)	7.80 (2.72)

The age distribution of our study cohort is right-skewed, with half of participants aged 26 or younger. Nearly two-thirds (59%) of our sample were men, 37% women and 3% (*n* = 9) neither male nor female. The majority had at least school-leaving academic qualifications. Non-binary study participants were included in all analyses, except for DIF. However, because of small sample size, their characteristics are not presented in Table 1. The proportion of the sample scoring above the GHQ-12 clinical cut-off point (Likert scoring method) was higher than has been reported for other studies using this measure (80% vs ~ 40%) (Moss et al. 2015; Angelo Picardi et al. 2015). There were no statistically significant differences between the GHQ-12 total scores between the male and female study participants.

### The Measure

The GHQ-12 contains 6 positively-phrased (PP) and 6 negatively-phased (NP) questions, asking the respondent to rate the degree to which they have experienced a symptom during the last week on a 4-point-scale (PP: better than usual, same as usual, less than usual, much less than usual; NP: not at all, no more than usual, rather more than usual, much more than usual). There are three scoring methods commonly used in the literature: the Standard GHQ scoring (or the binary method) (0011) aims to identify the presence vs absence of psychological distress by assigning 0 to the first two answer categories and 1 to the second two.

Goodchild and Duncan-Jones (1985) noted that using the second response category (not more than usual) for the negatively phrased questions could be interpreted as an admission of a chronic problem instead of the absence of one. Hence, the corrected GHQ scoring (or the Chronic method) assigns scores of 0111 for the 4-point answer categories of the NP questions and scores of 0011 for the PP questions. The Likert scoring method assigns 0123 to each response category.

### Consulting Autistic Adults About the Measure

The project’s Advisory Group, comprising twelve autistic adults without learning disabilities, were consulted on the project’s proposed measures, including the GHQ-12. This was done during a face-to-face meeting with the group, with copies of measures circulated to members in advance of the meeting. The meeting was facilitated by two members of the research team, who took turns to take detailed notes of the discussion. Both members agreed the final version of the notes of the meeting. They were then circulated to the Advisory Group and agreement with the notes confirmed at the following meeting.

### Statistical Analyses

#### Acceptability

In addition to consultation work with autistic adults described above, we assessed the acceptability of the GHQ-12 by examining missingness (Mack et al. 2018). Systematic patterns of missing data may indicate that some participants were less likely to answer specific questions than others, or that participants consistently avoided particular questions. This sort of missingness can be regarded as an indicator of the acceptability of a measure and can lead to biased results. We examined response rates across the 12 GHQ items (using Likert scoring method), looking for any systematic patterns of missing values across individual questions and we also examined the characteristics of participants who had any missing answers on the 12 items.

#### Reliability

We used Spearman item-total correlation to assess item discrimination. High correlation between an item and the overall score indicates good discriminatory power. We used Cronbach’s alpha to assess the reliability of the scale, i.e. that each item in the questionnaire consistently measures the same latent construct. The Kaiser–Meyer–Olkin Measure (KMO) was used to verify the sampling adequacy for the analysis. KMO values range from 0 to 1, indicating the proportion of variance in the variables that might be caused by underlying factors. KMO values between 0.8 and 1 indicate that the sampling is adequate.

#### Construct Validity

We conducted EFA across all three scoring methods of the GHQ-12, extracting factors using the Principal Axis Factoring (PAF) method. This extracts factors based on the covariance matrix which contains communalities, which are the squared multiple correlation between a measure and the other measures. Communalities reflect the variance in each measure due to the unique factor and random errors. PAF has been shown to be more accurate in reproducing population loadings than Principal Components Analysis (PCA) and is the preferred method for factor extraction (Russell 2002; Widaman 1993). We used Promax oblique rotation after factor extraction, allowing the rotated factors to be correlated. Only factors with an eigenvalue greater than 1 were considered significant. In addition, the scree plot was examined for inflexions indicating distinct factors. Items with a rotated factor loading of at least 0.4 and no cross-loading to other factors were retained for further modelling (Tabachnick & Fidell, 2014).

Confirmatory Factor Analysis (CFA) (based on Structural Equation Modelling, SEM) uses maximum likelihood estimation techniques to evaluate how well a hypothesized factor structure fits the observed data. We used the results of the EFA (assuming an oblique factor structure) to build a hypothesised model for each scoring method. The CFA performs a Chi-square goodness of fit test where non-significant results indicate good model fit. In CFA, sample size and normality of the data have been shown to influence results (Hu and Bentler 1999). Hu and Bentler recommend a ‘two criteria’ strategy in evaluating model fit. First, the Standardized Root Mean Square Residual (SRMSR) needs to be under 0.08. Second, at least one of the following fit statistics need to be significant: Tucker-Lewis Index (TLI), Bollen’s Index (IFI), the Comparative Fit Index (CFI), the Centrality Index (MFI), the Relative Noncentrality Index (RNI) and the Root Mean Square Error of Approximation (RMSEA). For TLI, IFI, CFI and RNI the criterion is 0.95 or greater. For MFI, the criterion is 0.90 or greater and for RMSEA, the criterion is 0.06 or lower (Russell 2002; Hu and Bentler 1999).

EFA and CFA were carried out using Stata version 15.0 software (Statacorp, College Station, TX). All *p *values are two-sided and set to *α* = 0.05 (Ayres et al. 2018).

#### Internal Consistency: Rasch Analysis

Rasch models are Item-response Models that help establish the internal consistency and reliability of a set of items. They assume that given a scale containing several items of different levels of ‘difficulty’ (in this case, severity of symptom of psychological distress), responses to these items can be predicted from the measured trait of individual respondents (referred to as ‘person ability’ and, in this case, mental health status).

An ordinal scale is converted to an interval scale by plotting individual items’ difficulty against respondents’ ability/trait severity. The model predicts an individual’s response based on the difference between their estimated score on the measured trait’s severity (e.g. depression) and the item’s difficulty. The predicted probability is measured in Log-Odds Units or Logits. The model uses Maximum Likelihood estimates of the person ability and the item difficulty levels based on the observed values so as to minimise response.

Assumptions of the Rasch model are a stochastic ordering of the items, local independence, unidimensionality and invariance [measured by Differential Item Functioning (DIF)]. The stochastic ordering of items entails parameter separation, whereby item difficulty and person ability do not depend on each other. Item ordering (from easiest to hardest to confirm) should be the same irrespective of ‘person ability’. Local Independence requires that affirming one item in the scale does not entail an affirmative answer to another item. The unidimensionality criteria entails that any subset of items should give the same estimate of person ability as any other subset if the scale is to measure ability on a linear interval scale. Invariance refers to how the scale performs in different populations. If the items function differently for different subpopulations (DIF) that indicates item bias.

We used the Rasch dichotomous model for the Standard and Chronic scoring methods, and the rating scale model for polytomous data for the Likert scoring method, entering all 12 items into the Rasch Model. We evaluated unidimensionality by using the T-test protocol, which tests whether there is significant difference in the person estimates between the two most different subsets of items identified within the first Principal Component extracted. If more than 5% of T-tests fall outside of the acceptable significance range that signifies multidimensionality in the data. Item Fit assesses the degree of divergence or residual between the expected value and the observed value for each person-item when summed over all items for an individual. Misfitting items have an absolute fit residual value of over 2.5 (Hendriks et al. 2012). Differential Item Functioning was tested by performing an ANOVA for each item, comparing scores across respondent characteristics (i.e.: gender and age groups). Uniform DIF is indicated by a significant p-value for main effect for person characteristic and it shows that one group of people displays a consistently greater ability to confirm an item than the other. Non-uniform DIF is indicated by a significant p value on the interaction effect and it shows that the ability to confirm an item is inconsistent across groups. Local Independence was tested by examining correlations between the residuals of the items. We defined an item pair as locally dependent if they had a residual correlation that was 0.2 higher than the total item residual average (Chen and Thissen 1997). We also explored changes in item location (i.e. difficulty ordering) across the 3 scoring methods. Rasch analysis was carried out using RUMM 2030.

#### Longitudinal Validity: Responsiveness to Change

Responsiveness to change is a component of validity. We defined responsiveness as longitudinal validity, i.e. the ability of the GHQ-12 to measure minimal important changes in mental health over time (Terwee et al. 2003). In order to evaluate longitudinal validity, we used the correlation approach suggested by Husted et al. (2000), and took responsiveness as the extent to which changes in one measure correspond to changes in a reference measure, measuring (approximately) the same outcome. We therefore examined the extent to which changes in the GHQ-12 between entry to the service and 12 months follow-up, correlated with changes in two other outcome measures: the WHO-QoL BREF psychological domain and the EQ5D-5L.

## Results

### Acceptability

The project’s Advisory Group of autistic adults without learning disabilities expressed no concerns about the GHQ-12. They did not consider any item to be offensive. Indeed of the mental health/well-being outcomes measures presented to the group for review (others being WHOQoL-BREF and the Short Warwick Edinburgh Mental Well-being Scale (Stewart-Brown et al. 2009), this was the most preferred. The group particularly liked the fact the measure asked about symptoms in relation to what was normal for them, rather than appearing to make assumptions about what is normal. It is, perhaps, useful to note here that another proposed measure was rejected by the group: evidence that the group felt empowered to express their opinions.

Our second indicator of acceptability was missingness. Missing data on the GHQ-12 was observed in just 5/308 study participants. Among these, 3 missed one question and 2 did not answer 2 questions out of the 12. There was no regular pattern in the items missed. All 5 respondents were male, but they had different socio-demographic characteristics.

### Exploratory Factor Analysis

The Spearman item-total correlation coefficients were significant for each item across every scoring method at *p* < 0.01 (Table 2). Item 2, ‘lost sleep’ had the lowest Spearman correlation coefficient across the Likert and the Standard scoring, whilst item 6, ‘overcome difficulties’ had the lowest coefficient on the Chronic scoring, followed by item 4, ‘making decisions’. The item-total correlation coefficients were overall highest when using the Likert scoring which was also reflected in the highest Cronbach’s alpha value for this scoring method compared to other scoring methods. All three scoring methods showed good internal consistency, reflected in α-values of 0.80 and above (Table 2).

**Table 2:** Item-total correlations and Cronbach’s alpha coefficients

	Likert	Standard	Chronic
1. Concentration	0.65	0.63	0.64
2. Lost sleep	0.58	0.53	0.41
3. Play useful part	0.62	0.60	0.63
4. Making decision	0.60	0.58	0.63
5. Under strain	0.71	0.64	0.39
6. Overcome diffs	0.73	0.68	0.32
7. Enjoy daily activities	0.68	0.64	0.68
8. Face up to problems	0.68	0.64	0.66
9. Feeling depressed	0.78	0.72	0.40
10. Losing confidence	0.80	0.69	0.46
11. Feeling worthless	0.75	0.68	0.52
12. Reasonably happy	0.72	0.68	0.69
α-coefficients	0.91	0.87	0.80

The Kaiser–Meyer–Olkin Measure (KMO) indicated good sampling adequacy for each scoring method (Likert KMO = 0.94, Standard KMO = 0.92, Chronic KMO = 0.85). For each scoring method, exploratory factor analysis (EFA) extracted 2 factors. Table 3 presents the rotated factor loadings. Standard scoring produced the best model, with 86% of the variance explained. Item 2 ‘lost sleep’ loaded weakest for the Likert and Standard scoring methods, whilst item 6, ‘overcome difficulties’ loaded weakest on the Chronic scoring method. The negatively phrased items 9 ‘feeling depressed’, 10 ‘losing confidence’ and 11 ‘feeling worthless’ seem to form the core of Factor 2 for Likert and Chronic methods. The results indicate that, for all scoring methods, there is moderate correlation between Factor 1 and Factor 2 with Likert scoring producing the strongest correlation (rho = 0.57).

**Table 3:** Rotated factor loadings of exploratory factor analysis, and fit statistics for confirmatory factor analysis, AS population (n = 303)

	Likert scoring		Standard scoring		Chronic scoring	
	Factor 1	Factor 2	Factor 1	Factor 2	Factor 1	Factor 2
1. Concentration	0.54		0.53		0.56	
2. Lost sleep		*0.33*	*0.35*			0.40
3. Play useful part	0.34			0.46	0.47	
4. Making decisions	0.56		0.37		0.57	
5. Under strain	0.49		0.52			0.45
6. Overcome difficulties	0.45		0.48			*0.38*
7. Enjoy daily activities	0.64		0.63		0.63	
8. Face up to problems	0.55		0.44		0.58	
9. Feeling depressed		0.59	0.45			0.57
10. Losing confidence		0.73		0.64		0.70
11. Feeling worthless		0.79		0.74		0.55
12. Reasonably happy	0.50		0.61		0.62	
Variance explained	0.80	0.77	0.86	0.74	0.76	0.67
Correlation between factors	0.57		0.55		0.43	

Italic value indicates rotated factor loading is less than 0.4

### Confirmatory Factor Analysis

For each scoring method, correlated two factor Structural Equation Models were built, including only those items that had rotated factor loadings of 0.4 or above. Model fit was evaluated, followed by an examination of the Modification Indices. Modification Indices provide information about omitted paths in the fitted model which would significantly improve model fit. We modified our original models, including paths which suggested significant improvement in the Chi-Square value.

**Fig. 1:**
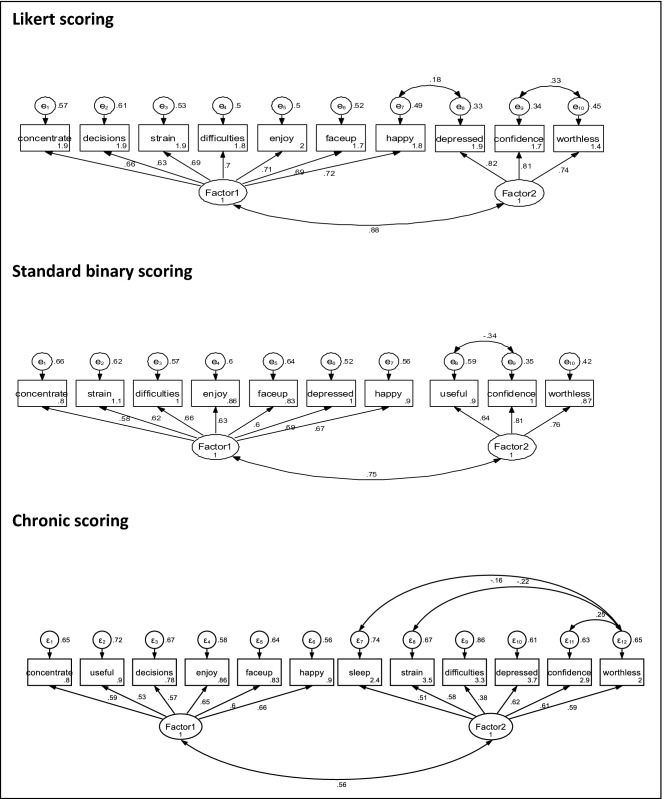
SEM models incorporating modification indices

Figure 1 presents the modified models, allowing for correlating error terms across selected items (based on the Modification Indices). The diagrams indicate that for the Likert and Chronic scoring method, items 9, 10 and 11 (‘depressed’, ‘confidence’ and ‘worthless’) form the core of the 2nd factor, with ‘confidence’ and ‘worthless’ displaying significantly correlating error terms. The model of the Standard scoring method shows a slight variation in the composition of the 2^nd^ factor, with item 3 ‘useful’ replacing item 9 ‘depressed’ and retaining items 10 and 11 (‘confidence’ and ‘worthless’). The factor structure of the Chronic scoring method seems to split the items into positively phrased (factor 1) and negatively phrased (factor 2) groups, a finding that replicates previous studies. Several of the negatively phrased items display significant degree of correlated errors, especially with item 11 ‘worthless’.

Chi-square values and fit index values for all models are presented in Table 4. The results suggest that the 2-factor solution produces satisfactory model fit across every scoring method, which can be significantly improved upon, incorporating correlating error terms based on the Modification Indices. The Standard scoring method has the best unmodified model fit (the Likert, the weakest), whilst both Standard scoring and Chronic scoring have excellent model fit, especially when allowing for correlating error terms on specific items.

**Table 4:** Different scoring methods: Factors found in EFA

	Likert scoring		Standard scoring		Chronic scoring	
SEM	EFA model	Modified*	EFA model	Modified*	EFA model	Modified*
P (χ^2^, df)	0.004 (59.64, 34)	0.248 (37.03, 32)	0.083 (45.93, 34)	0.394 (34.55, 33)	0.022 (75.61, 53)	0.706 (44.16, 50)
SRMSR (< 0.08)	0.033	0.025	0.35	0.29	0.042	0.032
BIC	6451.9	6440.7	3585.9	3580.2	2948.26	2933.95
CFI (> 0.90)	0.982	0.996	0.987	0.998	0.969	1.000
TLI (> 0.95)	0.976	0.995	0.983	0.998	0.961	1.011
RMSEA (< 0.06)	0.050	0.023	0.034	0.012	0.038	0.000

^*^Modified Model incorporates correlated error terms, if MI index >  = 10

We reanalysed our data using the Response Bias Model proposed by Hankins, which treats the GHQ-12 as a unidimensional measure with correlated error terms on all the negatively phrased items. The results are presented in Table 5. The Chronic scoring method shows the best fit to our data, producing very similar results to our 2-factor modified Chronic model.

**Table 5:** Different scoring methods: Hankins’ Response Bias Model*

	Likert	Standard	Chronic
P (χ^2^, df)	0.0045 (65.93, 39)	0.0034 (67.08, 39)	0.7410 (32.96, 39)
SRMSR (< 0.08)	0.029	0.035	0.027
BIC	7820.7	4394.324	2985.6
CFI (> 0.90)	0.984	0.974	1.000
TLI (> 0.95)	0.972	0.957	1.014
RMSEA (< 0.06)	0.048	0.049	0.000

^*^Response Bias Model: All 12 items assumed to form a unidimensional factor with correlating error terms on the negatively phrased questions

### Rasch Analyis

#### Dimensionality

We tested the dimensionality of the GHQ-12 index for each scoring method. Rasch analysis indicated that the GHQ-12 is not unidimensional (percentage < 5% = 8.03%) when the Likert scoring is used, but Standard scoring and Chronic scoring both produced unidimensional scales (Percentage < 5%: 0.00% and 2.61% respectively).

The GHQ-12 using the Likert scoring method showed three misfitting items with fit residuals over ± 2.5 (Table 6). Item 2 ‘lost sleep’ had a high positive fit residual indicating underdiscrimination, which suggests that this item does not measure the same concept as the other items on the scale. Items 9 and 10 (‘feeling depressed’ and ‘losing confidence’) had high negative residuals, indicating overdiscrimination. This is an indicative of redundancy. The Standard scoring method also indicated misfit for item 2 ‘lost sleep’, whilst there were no misfitting items when Chronic scoring was used.

**Table 6:** Item location and fit residuals

	Likert		Standard		Chronic	
	Item location	Fit residual	Item location	Fit residual	Item location	Fit residual
Concentration	− 0.01	0.11	0.39	0.50	1.88	− 0.65
Lost sleep	0.35	*3.46*	0.28	*3.10*	− 1.13	1.07
Play useful part	− 0.23	1.16	0.02	1.28	1.53	0.51
Making decisions	0.19	0.58	0.51	1.11	1.98	− 0.19
Under strain	− 0.35	− 0.26	− 0.60	− 0.22	− 2.39	− 1.34
Overcome difficulties	− 0.19	− 0.67	− 0.38	− 1.10	− 1.92	0.92
Enjoy daily activities	0.10	− 0.11	0.20	0.06	1.68	0.57
Face up to problems	0.18	0.05	0.29	0.05	1.76	− 0.79
Feeling depressed	− 0.28	*− 2.46*	− 0.43	− 1.74	− 2.62	− 1.68
Losing confidence	− 0.14	*− 3.34*	− 0.42	− 1.54	− 1.68	− 1.52
Feeling worthless	0.37	− 0.03	0.15	− 1.27	− 0.59	− 0.36
Reasonably happy	0.01	− 1.07	0.01	− 0.96	1.50	− 1.20

Italic indicates it residual > |2.5|

#### Local Dependency

The presence of local dependency indicates that a particular response to one item will predict the response to another item. Dependency between items has an impact on the dimensionality of the item set. We examined the person-item residual correlation matrix to identify dependency across items. Across each scoring method we found significant local dependency between items 10 and 11 (‘losing confidence’ and ‘thinking of self as worthless’).

#### Item Ordering

Ordering of items in terms of difficulty varied between scoring methods (Appendix 2). Items 10 and 2, ‘feeling worthless’ and ‘losing sleep’ were least likely to be affirmed when using the Likert scoring, yet they both moved up the order of difficulty when the Standard or Chronic scoring method was used. On the other hand, items 1 and 3 (‘being able to concentrate’ and feeling that one ‘plays a useful part’) were relatively problematic items with the Likert scoring method, but less problematic when Classic and Chronic scoring methods were used.

#### Differential Item Functioning

We used Rasch analysis to test for Differential Item Functioning by gender and age group (21 and under, 22–33 and 34 and over). Using the Likert and Chronic scoring methods, item 1 (‘able to concentrate’ *p* < 0.05) showed non-uniform DIF for gender. Using the Likert and Standard scoring, item 10 ‘losing confidence in self’ indicates non-uniform DIF (*p* < 0.05), where women were consistently more likely to affirm this item than men with the same level of depression (the difference is most marked among women and men with the lowest levels of psychological distress). We found no DIF across age-groups.

#### Responsiveness to Change

In the subsample of study participants who received specialised autism support (*n* = 252) we found significant correlation between the average change in the GHQ-12 (Likert scoring) and the average change in the WHO-QoL BREF psychological domain (*r* = 0.44, *p* < 0.001). Similarly, there was significant correlation between the average change in the GHQ-12 and the average change in the EQ5D-5L score (*r* = 0.43, *p* < 0.001). Change was measured between entry into the service and 12 months after leaving the service.

## Discussion

This paper reports an assessment of the psychometric properties and acceptability of the GHQ-12 for use with autistic adults without learning disability. The study was carried out in response to emerging concerns about untested use of mental health, and other measures developed for neurotypical adults with autistic adults. We used statistical methods to investigate the acceptability of the measure, the scale’s reliability, construct validity, internal consistency and responsiveness to change. Our findings indicate that the GHQ-12 performed well in this population with high response rates (98.4% of the population answering 12 out of 12 items). Moreover, the scale had good psychometric properties overall, with slight differences across scoring methods. These findings provide first evidence supporting the use of the GHQ-12 with populations of autistic adults without LD. We looked at how the measure performed in a sample of individuals referred to autism-specialist community-based services providing diagnostic assessment and mental health, social care and other interventions. Average GHQ-12 scores suggest a higher level of mental health difficulties than observed in community samples. Findings suggest that, for at least some of the population of autistic adults, researchers and clinicians can be confident that the scale is a reliable measure of mental health, and that comparing the GHQ-12 scores of autistic and neurotypical populations is legitimate and meaningful. However, further studies are needed to establish if this measure performs equally well when used with community samples not seeking autism-specialist support for mental health or other needs; or in tertiary/in-patient mental health settings. In addition, and importantly, we presented evidence indicating that autistic adults find this an acceptable measure to complete. The following sections discuss our findings, and provide guidance regarding the application of the scale’s different scoring methods.

### Factor Structure

In our sample of autistic adults without LD, the GHQ-12 showed a two-dimensional correlated factor structure for each scoring method, aligning with the findings of several previous studies of non-autistic populations (Toyabe et al. 2007; Gelaye et al. 2015; Hu et al. 2007). Further examination of our models, using CFA and Rasch analysis however, revealed the underlying causes of these seemingly multifactorial solutions. First, CFA showed that there are correlating error terms on at least some of the negatively phrased questions. Second, our Rasch analysis revealed significant local dependence across items 9 and 10, which form the core of the second factor, traditionally associated with the ‘depression/anxiety’ dimension. However, whether the GHQ-12 is regarded as a multidimensional index consisting of two correlating components, or as a unidimensional index with significant response bias on the negatively worded questions, is of no empirical relevance. This is because, for our study population, we found that, using the Chronic scoring method, the two-dimensional model—incorporating correlating error terms on some of the negatively phrased items performed equally well to the unidimensional Response Bias Model with correlating error terms on all negatively worded questions.

### Scoring Method Matters

The scoring method used has been found to affect the sensitivity (Crockett et al. 2009), discrimination (Hankins 2007) and dimensionality of the GHQ-12 (Martin and Newell 2005). Our study confirms this is also the case when the study population is autistic adults without LD. We found that the Standard scoring method explained the highest proportion of variance in the data, whilst the Chronic method produced the best modified two-dimensional and the best unidimensional Response Bias Model. The Likert method produced the smoothest distributions and highest Cronbach’s Alpha, but it was poorest at producing a unidimensional model.

Previous evaluations of the psychometric properties of the GHQ-12 have concluded that choice of scoring method should be informed by the research aim and study population. The Likert method is regarded as being more sensitive to change than the Standard and Chronic methods because it generates wider and less skewed distribution of scores, thus making it more suitable for use in parametric statistics (Banks et al. 1980). The Chronic method has been shown to have the best discriminatory properties in clinical populations (Clarke et al. 1993), in populations with multiple comorbidities (Goldberg et al. 1998) or in populations who are expected to have sustained levels of mental health problems (Toyabe et al. 2007). In particular, using the Chronic method moderates response bias by accounting for responses confirming long-standing negative mood states (Hankins 2008a) including in community samples (Toyabe et al. 2007; Lundin et al. 2016). Our findings indicate that such conclusions also stand for autistic adults without LD.

### Item Difficulty Ordering Differs by Scoring Method

Examining the results of the Rasch analysis suggests that the main reason why scoring method affects dimensionality, at least for autistic adults without LD, is that each scoring method produces a different item difficulty ordering (Appendix 2). Gao et al. (2012) had similar findings when validating the GHQ-12 across the cancer trajectory. They found that the Likert method was most suitable for the general community, the Chronic method for cancer outpatients and the Standard method for palliative care patients.

Moreover, Gao et al (2012) found each method produced a different item difficulty ordering in their three samples. This implies that the different scoring methods measure a different ‘severity profile’. In other words, the importance attributed to certain items seems to shift as the severity/duration of mental distress progresses.

In our sample, we found that items which have relatively low ‘difficulty levels’ (i.e. are unlikely to be affirmed) on the Likert scale, are more likely to be affirmed when Standard scoring, and even more when Chronic scoring, methods are applied. For example, relatively low numbers of respondents reported significant sleep disturbances (low scores on the Likert/Standard scale) but most people with chronic mental health problems did report some sleep problems (Chronic scoring). This explains the redundancy (reduced explanatory power) of item 2 ‘lost sleep’ when using the Likert/Standard scoring methods, yet its increased relevance when using the Chronic scoring method (Table 6).

The Chronic scoring method therefore appears to be better at differentiating case severity, since it produces an item difficulty ordering that attributes higher weight to items that are often experienced by people undergoing chronic psychiatric distress. For example, two respondents with the same Likert total score can have different Chronic total scores depending on which items they confirmed. This finding indicates, that in populations where the presence of long-standing psychiatric distress is suspected, the Chronic scoring method is preferable.

### Limitations

This study was based on secondary data analysis of a dataset collected as part of a national evaluation of community-based autism-specialist services for autistic adults without LD. For this reason, it was not possible to include in the study design an assessment of the content or face validity of GHQ-12, or perform test-re-test reliability checks. Further work to assess these components of validity and test–retest reliability, is therefore required.

Our study population consisted of adults without LD referred to community-based autism-specialist services either for an autism-diagnostic assessment (and, if identified in the assessment process, mental health/social interventions) or, if already diagnosed with autism, mental health/social interventions. Not unexpectedly, compared to studies which have used non-clinical samples (Moss et al. 2015; Picardi et al. 2015), a greater proportion of our sample scored above the general population clinical cut-off point on the GHQ-12 (Beresford et al. in press). The study did not collect data on specific mental health diagnoses, and we were therefore unable to test whether a mental health diagnosis, and the nature of that diagnosis, affects the scale’s performance. In addition, the sample was skewed towards younger aged adults. It would be important, therefore, to replicate this study in a larger, non-clinical population of autistic adults without LD, and with increased representation of older individuals and those identifying themselves as non-binary. In addition, further work to ascertain the GHQs properties across specific mental health diagnoses would further strengthen our understanding of this measure, and inform its use.

## Conclusion

We assessed the acceptability and psychometric properties of the GHQ-12 when used with a population of autistic adults without LD. We found that the items and wording of the GHQ-12 was regarded as acceptable by a group of autistic adults without LD. The high completion rate observed in our study population provides further evidence of its acceptability. Moreover, it had good psychometric properties and functioned as a unidimensional scale with some correlation across the error terms of the negatively worded items. The Chronic scoring method produced the model with the best discriminatory power. We found that different scoring methods resulted in different item difficulty ordering which were associated with the factorial structure of the models. The GHQ-12 had good longitudinal validity, displaying correlation in change over time with other measures of psychological well-being. Our findings are similar to studies which have evaluated the psychometric properties of the GHQ-12 in non-autistic populations. They indicate that the GHQ-12 is a robust measure of psychological distress in autistic adults without LD, but care must be taken to choose the scoring method that is most appropriate for the study or clinic population.

## Appendix 1

See Appendix Table

**Table 7:** GHQ-12: positively phrased and negatively phrased questions

Positively phrased items	Negatively phrased items
Able to concentrate	Lost sleep over worry
Felt playing useful part in things	Felt constantly under strain
Felt capable of making decisions	Felt could not overcome difficulties
Able to enjoy day-to-day activities	Been feeling unhappy and depressed
Been able to face problems	Been losing confidence in self
Feeling reasonably happy	Been thinking of self as worthless

7.

## Appendix 2

See Appendix Table

**Table 8:** Item difficulty from most trouble-free item to most problematic item (increasing severity)

Likert	Standard	Chronic
1*. *Feeling worthless	1. Making decisions	1. Making decisions
2. Lost sleep	2. Concentration	2. Concentration
3. Making decisions	3. Face up to problems	3. Face up to problems
4. Face up to problems	4. Lost sleep	4. Enjoy daily activities
5. Enjoy daily activities	5. Enjoy daily activities	5. Play useful part
6. Reasonably happy	6. Feeling worthless	6. Reasonably happy
7. Concentration	7. Play useful part	7. Feeling worthless
8. Losing confidence	8. Reasonably happy	8. Lost sleep
9. Overcome difficulties	9. Overcome difficulties	9. Losing confidence
10. Play useful part	10. Losing confidence	10. Overcome difficulties
11. Feeling depressed	11. Feeling depressed	11. Under strain
12. Under strain	12. Under strain	12. Feeling depressed

8.
